# Bag-1 stimulates Bad phosphorylation through activation of Akt and Raf kinases to mediate cell survival in breast cancer

**DOI:** 10.1186/s12885-019-6477-4

**Published:** 2019-12-28

**Authors:** Tugba Kizilboga, Emine Arzu Baskale, Jale Yildiz, Izzet Mehmet Akcay, Ebru Zemheri, Nisan Denizce Can, Can Ozden, Salih Demir, Fikret Ezberci, Gizem Dinler-Doganay

**Affiliations:** 1 Department of Molecular Biology and Genetics, Istanbul Technical University, 34469 Istanbul, Turkey; 2 Department of Pathology, Umraniye Teaching and Research Hospital, 34764 Istanbul, Turkey; 3 Department of General Surgery, Umraniye Teaching and Research Hospital, 34764 Istanbul, Turkey

**Keywords:** Bag-1, Bad phosphorylation, Akt, Raf kinase, Breast cancer, Apoptosis, Cell survival

## Abstract

**Background:**

Bag-1 (Bcl-2-associated athanogene) is a multifunctional anti-apoptotic protein frequently overexpressed in cancer. Bag-1 interacts with a variety of cellular targets including Hsp70/Hsc70 chaperones, Bcl-2, nuclear hormone receptors, Akt and Raf kinases. In this study, we investigated in detail the effects of Bag-1 on major cell survival pathways associated with breast cancer.

**Methods:**

Using immunoblot analysis, we examined Bag-1 expression profiles in tumor and normal tissues of breast cancer patients with different receptor status. We investigated the effects of Bag-1 on cell proliferation, apoptosis, Akt and Raf kinase pathways, and Bad phosphorylation by implementing ectopic expression or knockdown of Bag-1 in MCF-7, BT-474, MDA-MB-231 and MCF-10A breast cell lines. We also tested these in tumor and normal tissues from breast cancer patients. We investigated the interactions between Bag-1, Akt and Raf kinases in cell lines and tumor tissues by co-immunoprecipitation, and their subcellular localization by immunocytochemistry and immunohistochemistry.

**Results:**

We observed that Bag-1 is overexpressed in breast tumors in all molecular subtypes, i.e.*,* regardless of their ER, PR and Her2 expression profile. Ectopic expression of Bag-1 in breast cancer cell lines results in the activation of B-Raf, C-Raf and Akt kinases, which are also upregulated in breast tumors. Bag-1 forms complexes with B-Raf, C-Raf and Akt in breast cancer cells, enhancing their phosphorylation and activation, and ultimately leading to phosphorylation of the pro-apoptotic Bad protein at Ser112 and Ser136. This causes Bad’s re-localization to the nucleus, and inhibits apoptosis in favor of cell survival.

**Conclusions:**

Overall, Bad inhibition by Bag-1 through activation of Raf and Akt kinases is an effective survival and growth strategy exploited by breast cancer cells. Therefore, targeting the molecular interactions between Bag-1 and these kinases might prove an effective anticancer therapy.

## Background

Breast cancer is the most common cancer in women worldwide, and, despite advancements in early detection and treatment, it remains to be the leading cause of cancer-related deaths in women [1]. The multifunctional anti-apoptotic Bcl-2-associated athanogene (Bag-1) protein is overexpressed in multiple cancer types, including breast cancer, and is a potential drug target for cancer treatment [2–4]. Bag-1 interacts with a variety of proteins including Bcl-2, Raf-1 (also known as C-Raf), Akt, Hsp70/Hsc70, proteasome, certain growth factor receptors and nuclear hormone receptors (e.g., estrogen receptor, glucocorticoid receptor, etc.), as well as DNA. Through these interactions, Bag-1 is involved in the regulation of diverse cellular processes such as apoptosis, proteostasis, transcription and motility [5]. Bag-1 protects cells from a variety of apoptotic signals [2, 6], and plays important roles in breast cancer development and chemoresistance [7].

Bag-1 has three major isoforms; Bag-1 L (50 kDa), Bag-1 M (46 kDa) and Bag-1S (36 kDa). All isoforms contain a BAG domain and an ubiquitin-like (UBL) domain, which are important for binding to Hsp70 chaperones and the proteasome, respectively [8, 9]. Bag-1 isoforms differ at their N-termini. Bag-1 L contains a nuclear localization signal (NLS) and is predominantly found in the nucleus [10]. Bag-1 M and Bag-1S are mostly cytosolic, lacking an NLS, but they can also be transported into the nucleus under stress conditions [11]. Bag-1 L, but not the other isoforms, increases the transcriptional activity of estrogen receptor (ER), an important growth hormone for breast epithelial cells [12]. Hence, Bag-1 isoforms might assume different cellular functions and localizations.

Bag-1’s interactions with the proteins involved in growth factor-dependent signaling pathways are important for its anti-apoptotic, pro-survival function. C-Raf is a serine/threonine kinase in the Ras signaling pathway, which is frequently activated in cancer [13]. Interaction of Bag-1 with C-Raf activates C-Raf and downstream ERKs, independent of Ras activation in cell culture [14, 15]. Bag-1 also binds to both B-Raf and Akt, and a tripartite complex formation has been postulated [16]. Akt kinase, downstream of PI-3 K, phosphorylates the pro-apoptotic Bad protein specifically at Ser-136 [17]. Phosphorylation of Bad creates a binding site for 14–3-3 proteins, which sequester Bad in the cytosol preventing its apoptotic mission at the mitochondrial membrane [18]. Studies in Bag-1 knockout mice showed that Bag-1 is essential for phosphorylation of Bad at Ser-136, but not at Ser-112 and Ser-155, and for inhibition of apoptosis in hematopoietic and neural stem cells [16].

In this study, we aimed to understand in more detail Bag-1’s pro-survival mechanism in breast cancer. We first tested whether Bag-1 was overexpressed in all molecular subtypes of breast cancer. Then we addressed whether Bag-1-induced survival was mediated through activation of Akt and Raf pathways, and inhibition of Bad function. We investigated how the expression and phosphorylation of B-Raf, C-Raf, Akt and Bad changed upon Bag-1 overexpression and knockdown in breast epithelial and breast cancer cell lines. We also investigated expression levels of these proteins in tumor and normal tissues from breast cancer patients with different molecular subtypes to understand if these pro-survival mechanisms have potential clinical basis. Our findings supported the importance of Bag-1-mediated Akt and Raf activation and Bad inhibition in breast cancer etiology.

## Methods

### Cell culture

MCF-7 (ATCC® HTB-22™), BT-474 (ATCC® HTB-20™), and MDA-MB-231 (ATCC® HTB-26™) human breast cancer cells were grown in DMEM containing 25 mM glucose, 1 mM sodium pyruvate and 4 mM L-glutamine (Gibco- Invitrogen), supplemented with 10% fetal bovine serum, 100 units/ml Penicillin and 100 μg/ml Streptomycin (Pan Biotech). MCF-10A (CRL-10317, ATCC) human breast epithelial cells were grown in DMEM/F12 (1:1) medium (Gibco-Invitrogen), supplemented with 10% horse serum (Invitrogen), 100 units/ml Penicillin and 100 μg/ml Streptomycin, 2.5 mg/ml insulin (Invitrogen), 150 μg/ml cholera enterotoxin (Sigma-Aldrich), 2.5 mg/ml hydrocortisone (Sigma-Aldrich) and 20 ng/ml epidermal growth factor (Sigma-Aldrich). Cells were maintained at 37 °C in a 5% CO_2_ humidified atmosphere.

### Breast tissue samples

Breast tissue samples were obtained from 30 female breast cancer patients, with a mean age of 52 years, who were recruited in Umraniye Training and Research Hospital (UEAH) in Istanbul between 2015 and 2017. All patients gave their informed consent for usage of their specimen for research. The study was approved by the ethical committee at the Department of General Surgery of UEAH. Patients did not receive any chemotherapeutic treatment before surgery. Fresh primary tumor tissues and normal breast tissues from the same patients were taken from dissection material after surgery (mastectomy and lumpectomy). Molecular subtypes of tumor samples were graded by an experienced pathologist. Normal breast tissue was taken 1 cm away from the tumor. Approximately 0.5 cm^3^ of tissue samples were spared for storage. A fraction of the tissue was immediately transferred into 1 ml RNA Later Tissue Stabilization Solution (Ambion) for short term storage at − 20 °C, while the rest was formalin-fixed for 24 h and paraffin-embedded (FFPE) for long term storage.

### Plasmid and antibodies

Bag-1 expression plasmid was generated by Capital Biosciences (MD, USA). C-terminal TAP (tandem affinity purification) tag was added to human Bag-1 cDNA ORF after removing the stop codon, and the construct was cloned into pEZ-M02 vector (GeneCopoeia, MD, USA).

All antibodies were purchased from Cell Signaling Technology. The monoclonal primary antibodies were mouse α-Bag-1, rabbit α-β-actin, rabbit α-Bcl-2, rabbit α-Bax, rabbit α-Bad, rabbit α-Phospho-Bad (Ser136), rabbit α-Phospho-Bad (Ser112), rabbit α-C-Raf, rabbit α-Phospho-C-Raf (Ser338), rabbit α-B-Raf, rabbit α-Phospho-B-Raf (Ser445), rabbit α-A-Raf, rabbit α-14-3-3, rabbit α- Akt, rabbit α-Phospho-Akt (Ser473), rabbit α-Hsp70 and rabbit α-Vinculin.

### Transient transfection

50–70% confluent cells in 6-well plates were transfected with Bag-1 expression plasmid using X-tremeGENE HP DNA transfection reagent (Roche) or with siRNAs using HiPerFect transfection reagent (Qiagen), according to the manufacturers’ protocols. siRNAs were used to silence the expression of Bag-1 (sc-29,211, Santa Cruz), Raf-1 (sc-29,462, Santa Cruz), and B-Raf (#8935, Cell Signaling Technology). Negative control siRNAs (Qiagen) were also used. Cells were lysed 24 and 48 h after plasmid or siRNA transfection.

### Inhibitor treatment

MK-2226 dihydrochloride (Santa Cruz) and GW 5074 (Santa Cruz) were used to inhibit Akt and Raf-1, respectively. 5 × 10^5^ cells were seeded on 6-well plates. After incubation for 24 h, cells were treated with 0, 10, 150 and 500 nM of MK-2622, or 0, 10, 25 and 50 μM of GW-5074 for 24 h before protein extraction.

### XTT cell viability assay and apoptotic death assay

Cells were seeded at 1 × 10^4^ cells/well in quadruplicate in 96-well plates, and grown for 24 h before transfection. 24, 48 and 72 h after transfection, cell viability and apoptotic cell death was quantified using XTT Cell Viability Kit (Cell Signaling Technology) and Cell Death Detection ELISAPLUS kit (Roche), following the manufacturers’ protocols. Colorimetric measurements were done at 450 nm and 405 nm, respectively, using Benchmark Plus ELISA microplate reader (Bio-Rad). Cell viability and apoptotic death assays were repeated with three and two biological replicates, respectively.

### Cell lysis and immunoblotting

Pellets of 1 × 10^6^ cells were lysed on ice in ProteoJET mammalian cell lysis reagent (Fermentas), supplemented with cOmplete protease inhibitor cocktail (Roche) and PhosSTOP phosphatase inhibitors (Roche). Cell lysates were centrifuged at 20000 *g* for 20 min at 4 °C, and supernatants were taken to new tubes. Protein concentration was determined by Bradford assay (Fermentas). 10 μg proteins from each sample were fractioned on 12% SDS-PAGE, and transferred to a nitrocellulose membrane using Trans-Blot Turbo transfer system (Bio-Rad). Membranes were blocked in 5% BSA TBS-Tween20, washed, and incubated with the primary antibody (1:500 for all, except 1:1000 for anti-14-3-3) overnight at 4 °C. Membranes were washed again and incubated with the appropriate HRP-conjugated secondary antibody (sheep anti-mouse or goat anti-rabbit; Cell Signaling Technology, 1:5000) for 2 h. After the final wash step, membranes were treated with ECL substrate and imaged in ChemiDoc MP imaging system (Bio-Rad). Densitometric analysis was performed using Adobe Photoshop CS5 software.

### Protein extraction from tissues

Frozen tissue samples were grinded using pestle and mortar in liquid nitrogen, and suspended in T-PER tissue protein extraction reagent (20 mL per 1 g tissue; Thermo Scientific), supplemented with 2 mM PMSF, 0.01 mM sodium orthovanadate, 1x PhosSTOP (Roche) and 1x cOmplete Protease Inhibitor Coctail (Roche). The homogenates were centrifuged at 12000 *g* and 4 °C for 15 min, and the supernatants were incubated overnight at − 20 °C. Proteins were precipitated by centrifugation at 8000 *g*, 4 °C for 10 min. Pellets were washed with chilled acetone, centrifuged for 5 min at 10000 *g*, 4 °C. Pellets were dried for 5 min and resuspended in urea buffer (8 M urea, 2% CHAPS and 0.1% 2-mercaptoethanol). The suspension was incubated at 4 °C for 4 h and centrifuged for 5 min at 10000 *g* to remove any insoluble material. Protein concentration was measured with Bradford assay.

### Immunoprecipitation

Monoclonal anti-Bag-1 antibody was incubated with Dynabeads Protein G (Invitrogen) with rotation for 30 min at room temperature. Tissue and cell extracts were adjusted to 0.5 mg/mL total protein in appropriate lysis buffer and incubated with antibody-coupled beads overnight at 4 °C with rotation. The buffer was removed and immunocomplexes were eluted in 20 μl elution buffer (50 mM glycine, pH 2.8). 5 μl of 4X Laemmli buffer was added, and incubated for 10 min at 70 °C to dissociate the complexes and denature the proteins prior to fractionation in 12% SDS-PAGE.

### Immunocytochemistry

Cells were seeded as 2.5 × 10^4^ cells per well in 12-well plate containing a poly-L-lysine coated coverslip, and transfected with Bag-1 plasmid. After 48 h, culture medium was removed, and cells were washed twice with phosphate buffered saline (PBS) solution. Cells were fixed in prechilled methanol and incubated for 15 min at − 20 °C and washed three times with PBS. Non-specific binding was blocked by 1-h incubation in BSA blocking buffer (10% antibody specific serum, 10 mg/mL bovine serum albumin (BSA) in PBS). Cells were incubated with appropriate primary antibodies overnight at 4 °C. Primary antibodies used were mouse anti-Bag-1 (1:200), rabbit anti-β-actin (1:200), rabbit anti-C-Raf (1:200), rabbit anti-B-Raf (1:200), rabbit anti-Bcl-2 (1:200), rabbit anti-Hsp70 (1:200), rabbit anti-Phospho-Akt (Ser473) (1:200). Following washing, cells were incubated for 1 h at 37 °C with the secondary antibody (Alexa Flour® 647 goat anti-mouse or Alexa Flour® 488 goat anti-rabbit, Invitrogen, 1:1000 for both). After extensive washing, coverslips were mounted on slides using Vectashield mounting medium containing DAPI (Sigma-Aldrich). Confocal images were obtained under 63X magnification using Leica TCS SP2 SE confocal imaging system (Leica, Germany). For quantification of colocalizations, Pearson’s *r* was calculated by using Fiji plug-in of ImageJ.

### Immunohistochemistry

The paraffin-embedded tissue blocks were sectioned at a thickness of 4 μm and placed on slides. The slides were deparaffinized by two rinses of xylene, followed by two rinses of 100% ethanol. Antigen retrieval was done by heating the slides in a pressure cooker filled with 7.5 mM sodium citrate, pH 6.0. Slides were blocked in casein for 5 min to prevent non-specific antibody binding. To examine the density and localization of Bag-1, Akt, p-Akt, Bad, p-Bad-Ser112 and p-Bad-Ser-136 proteins, tissue sections were incubated with primary antibodies for 25 min. Detection was performed with Bond Polymer Refine Detection kit on Bond Max Autostainer (Leica Biosystems, UK). HRP-conjugated secondary antibodies and DAB (3,3′-diaminobenzidine) substrate were used to visualize the immunocomplexes via brown precipitates. Slides were counterstained with hematoxylin (blue) to visualize cell nuclei. The images were obtained using fluorescence microscope (Nikon, Eclipse; Camera: DS-Ri2). Nuclear and cytoplasmic staining positivity were scored from 0 to 100%, and staining intensity was scored from 1 to 3 (1: weak, 2: moderate, 3: strong). The final reactivity score was determined by dividing the product of positivity score and intensity score by 3 to have a range of 0 to 100. The result of immunostaining was designated negative if the reactivity score was below 10, weakly positive between 10 to 50, and strongly positive above 50.

### In silico predictions of protein-protein interactions

We used PRISM (Protein Interactions by Structural Matching) to predict the interactions between Bag-1, Akt, C-Raf and B-Raf proteins [19]. Protein structures were obtained from Protein Data Bank (PDB); 1HX1:B for Bag-1 BAG domain, 1WXV:A for Bag-1 UBL domain, 3OMV:A for C-Raf, 4MNE:B for B-Raf, and 4EKL:A for Akt. Predicted protein complexes were ranked by FiberDock according to their energies, and complexes with a binding energy score (BES) > − 10 were ignored. Predicted protein-protein interactions were further analyzed using PyMOL [20].

### Statistical analysis

All experiments were performed in triplicates unless otherwise specified; mean ± standard error of the mean (S.E.M.) were given. Statistical significance (*p* < 0.05) was determined by unpaired *t*-test assuming unequal variances, or by one-way or two-way ANOVA test using GraphPad Prism 6 (GraphPad Software, USA). *p* values were represented as following: *p < 0.05; **p < 0.01, ***p < 0.001, and ****p < 0.0001.

## Results

### Bag-1 expression in breast cancer cells confers increased cell survival

**Fig. 1:**
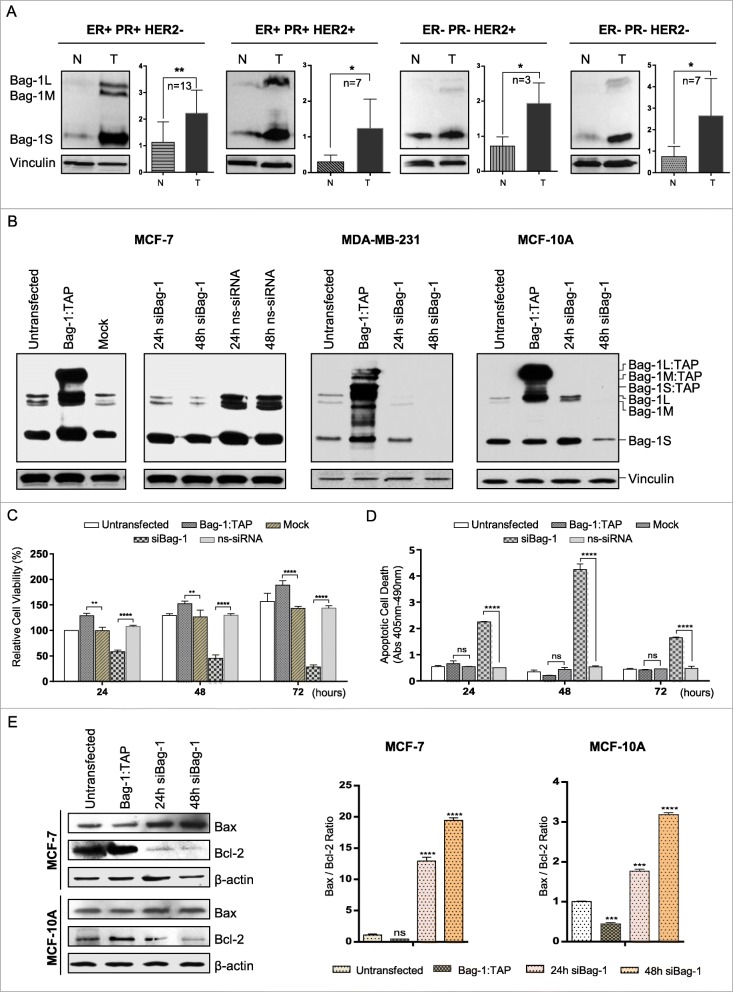
Bag-1 increases cell survival and suppresses apoptotic cell death. **a**. Immunoblot analysis of Bag-1 expression in normal (N) and tumor (T) tissues of 30 breast cancer patients with different expression profiles of ER/PR and Her2 receptors. Bag-1 expression was normalized to vinculin. **b**. Immunoblot analysis of Bag-1 protein levels in MCF-7, MDA-MB-231 and MCF-10A cells. Cells were transfected with Bag-1 (Bag-1:TAP) expression vector, mock vector, Bag-1 siRNA (siBag-1), or non-silencing control siRNAs (nc-siRNA). Expression of Bag-1 isoforms was detected with Bag-1 antibody. β-actin was used as the loading control. **c**, **d**. Cell viability and apoptotic cell death following Bag-1 overexpression or Bag-1 silencing in MCF-7 cells. XTT cell viability assay (**c**) and Cell Death Detection ELISAPLUS assay (**d**) was performed 24, 48 and 72 h after transfection of MCF-7 cells with Bag-1 expression vector, Bag-1 siRNA, and their negative controls. All values are given relative to 24 h untransfected control in XTT assay. Data are represented as mean ± standard error from three independent experiments for XTT assay and two independent experiments for apoptosis assay. Two-way ANOVA was used to calculate *p* values. **e**. Effects of Bag-1 expression on Bax/Bcl-2 ratio in MCF-7 and MCF-10A cells. Expression levels of pro-apoptotic Bax and anti-apoptotic Bcl-2 proteins were determined by densitometric analysis on the immunoblots of MCF-7 and MCF-10A cell lysates after Bag-1 overexpression (48 h) or Bag-1 silencing (24 and 48 h). Bax and Bcl-2 levels were normalized according to β-actin levels, and Bax/Bcl-2 ratio was calculated to determine the apoptotic potential of cells at each condition

Breast cancer patients are stratified into different molecular subtypes with respect to the expression of nuclear hormone receptors (i.e.*,* estrogen receptor (ER) and progesterone receptor (PR), and epidermal growth factor receptor (Her2). Four major subtypes are ER/PR + Her2-, ER/PR + Her2+, ER/PR-Her2+, and ER/PR-Her2- [21]. To examine how Bag-1 expression was altered in each of these subtypes, we performed immunoblot analyses to detect Bag-1 levels in normal and tumor tissues from 30 breast cancer patients with different subtypes. We found that Bag-1 expression was significantly higher in tumor cells than in normal cells in all subtypes (Fig. 1a, Additional file 1: Figure S1).

To assess the role of Bag-1 on cell viability and apoptosis, MCF-7 breast cancer cells were transfected with Bag-1 expression vector or Bag-1 siRNA (Fig. 1b), and cell viability (Fig. 1c) and apoptotic cell death (Fig. 1d) were monitored for a period of 72 h. XTT assay showed that Bag-1 overexpression significantly increased cell growth rates such that Bag-1-transfected cells were 32.9% more than mock-transfected cells after 72 h (*p* < 0.0001). On the other hand, Bag-1-silencing severely reduced cell viability; the number of cell transfected with Bag-1 siRNA was only 20.8% of the number of cells transfected with non-silencing siRNAs after 72 h (*p* < 0.0001). Cell Death ELISAPLUS assay showed that apoptotic cell death sharply increased in Bag-1-silenced cells (~ 4.5-fold, *p* < 0.0001), but not in wild-type or non-silencing siRNA (ns-siRNA)-transfected cells, after 24 h, and this increase became more profound after 48 h (~ 9-fold, *p* < 0.0001) (Fig. 1d). Similar results were found for non-tumorigenic MCF-10A breast epithelial cells (Additional file 1: Figure S2). Next we investigated the effects of Bag-1 expression on pro-apoptotic Bax and anti-apoptotic Bcl-2 protein levels in MCF-7 and MCF-10A cells by immunoblotting (Fig. 1e). Bag-1 silencing significantly increased Bax levels and lowered Bcl-2 levels, while Bag-1 overexpression resulted in the opposite pattern albeit weakly. Bax/Bcl-2 ratio determines the apoptotic potential of a cell [22]. Bag-1-silenced MCF-7 cells had ~13 and ~19-fold higher Bax/Bcl-2 ratios at 24 and 48 h compared to wild-type cells, indicating that they were highly susceptible to apoptosis. Bag-1-silenced MCF-10A cells also had a higher Bax/Bcl-2 ratio (~ 2-fold at 24 h and ~ 3-fold at 48 h), even though not to the same extent as MCF-7 cells. Altogether, Bag-1 downregulation rendered cells susceptible to apoptosis.

### Bag-1 overexpression activates Raf and Akt signaling pathways in breast cells

**Fig. 2:**
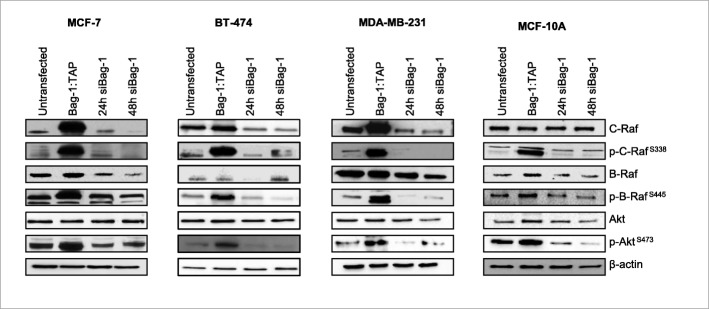
Bag-1 expression upregulates C-Raf, B-Raf and Akt. Cells were transfected with Bag-1 vector for 48 h or Bag-1 siRNA for 24 and 48 h. Total protein was analyzed by immunoblotting with specific antibodies against C-Raf, B-Raf, Akt, phospho-C-Raf^Ser338^, phospho-B-Raf^S445^ and phospho-Akt^S473^

Raf and Akt signaling pathways are commonly activated in cancer, promoting cell survival and growth. To understand whether Bag-1 overexpression or silencing affects these pathways in breast cell lines, we investigated the expression and phosphorylation levels of C-Raf, B-Raf and Akt proteins by immunoblot analysis of MCF-7, BT-474, MDA-MB-231 and MCF-10A cells upon Bag-1 overexpression or Bag-1-silencing (Fig. 2; Additional file 1: Figure S3). We observed that Bag-1 overexpression strongly increased C-Raf expression levels and its phosphorylation at Ser338. Bag-1 overexpression increased total B-Raf levels very subtly and did not alter total Akt levels at all, however it strongly increased phosphorylation of B-Raf at Ser445 and phosphorylation of Akt at Ser473. Hence, Bag-1 overexpressing breast cells activated both Raf and Akt pathways. We observed opposite effects upon Bag-1 silencing.

**Fig. 3:**
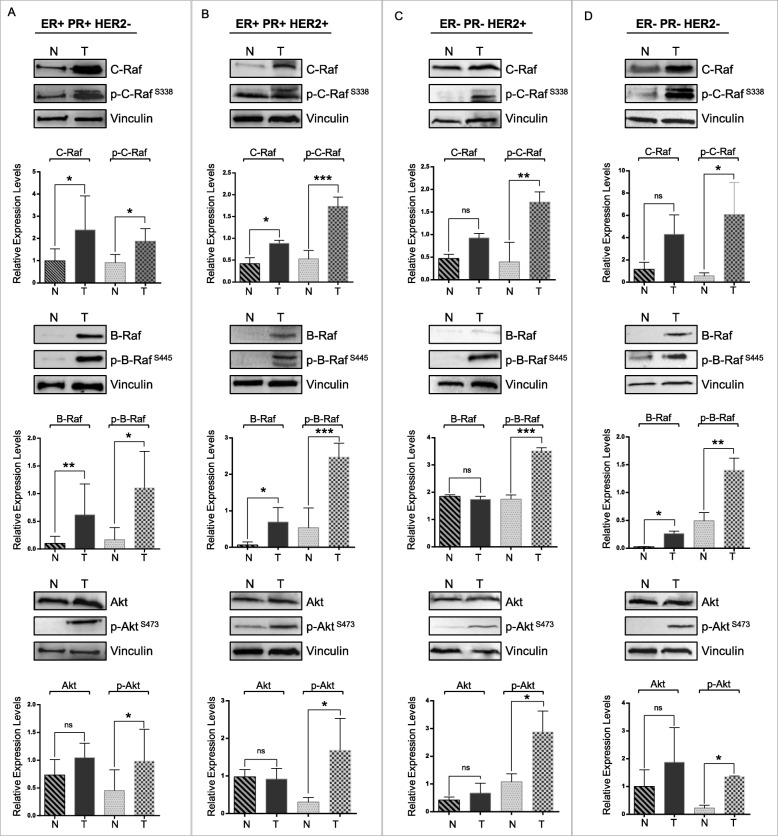
Raf and Akt pathways are activated in breast cancer. Immunoblots of C-Raf, B-Raf, Akt and their phosphorylated forms in tumor and normal tissues from breast cancer patients with four major molecular subtypes; **a**. ER + PR + Her2-, **b**. ER + PR + Her2+, **c**. ER-PR-Her2+ and **d**. ER-PR-Her2- breast cancer patients. Expression levels were analyzed with t-test after normalization to vinculin

Next, we assessed C-Raf, B-Raf, Akt expression and phosphorylation levels in tumor and normal tissues from ER/PR + Her2- (Fig. 3a), ER/PR + Her2+ (Fig. 3b), ER/PR-Her2+ (Fig. 3c) and ER/PR-HER2- (Fig. 3d) breast cancer patients by immunoblot analysis. C-Raf, B-Raf expression and phosphorylation levels were significantly increased in all tumor samples (Additional file 1: Figure S4, Additional file 1: Figure S5). Phosphorylation of Akt, but not its total protein level, also increased significantly in these tumors. Therefore, the effects of Bag-1 overexpression in cultured breast cells were in line with the observations in breast cancer patients in terms of C-Raf, B-Raf and Akt expression and activation dynamics.

### Bag-1 enhances the activity of Akt and Raf kinases to promote bad phosphorylation-dependent survival pathway

**Fig. 4:**
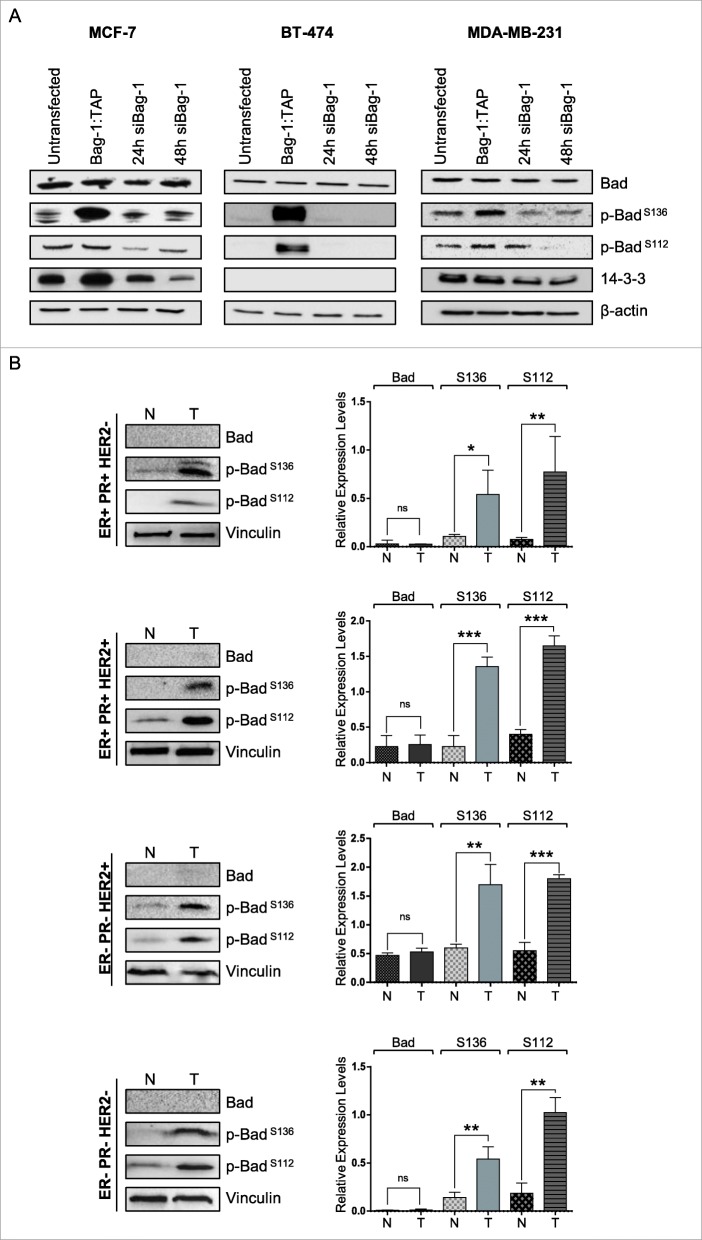
Bad phosphorylation at Ser136 and Ser112 increases upon Bag-1 overexpression. **a**. Immunoblot analysis of total and phosphorylated Bad levels in cell lysates from Bag-1 overexpressing, Bag-1-silenced and untransfected cells. **b**. Bad expression and phosphorylation profiles in tumor and normal tissues of breast cancer patients with ER + PR + Her2-, ER + PR + Her2+, ER-PR-Her2+ and ER-PR-Her2- subtypes. Expression levels were normalized to vinculin, and t-test was used to evaluate significant changes

Activated Raf and Akt kinases result in inhibition of pro-apoptotic Bad protein by phosphorylation at distinct residues (Ser112 and Ser136, respectively), leading to its sequestration by 14–3-3 proteins in the cytosol [23]. Inhibition of C-Raf by GW5074 and Akt by MK2226 in MCF-7 and MDA-MB-231 cells decreased Bad phosphorylation at Ser112 and Ser136, respectively, showing the importance of these kinases in Bad inhibition (Additional file 1: Figure S7). To better understand the mechanism of Bag-1-mediated cell survival in breast cancer cell lines, we investigated phosphorylation levels of Bad protein in MCF-7, BT-474 and MDA-MB-231 cells after transfection with Bag-1 plasmid or Bag-1 siRNA (Fig. 4a, Additional file 1: Figure S6). Although Bad expression remained unchanged, Bad phosphorylation at Ser136 and Ser112 was significantly increased upon Bag-1 overexpression, and significantly decreased upon Bag-1 silencing. Additionally, 14–3-3 protein levels also changed in parallel to Bag-1 levels, which might also reinforce Bag-1’s inhibitory effects on Bad inhibition. We also investigated total Bad and phospho-Bad levels in normal and tumor tissues of ER/PR + Her2-, ER/PR + Her2+, ER/PR-Her2+ and ER/PR-HER2- breast cancer patients (Fig. 4b). We observed that both Ser136 and Ser112 phosphorylation were significantly increased in tumor cells, in line with the observed increased levels and activities of Bag-1, Akt, and Raf in tumors. On the other hand, Bad levels did not increase significantly in tumors.

### Bag-1 forms complexes with Akt and Raf kinases in breast cells

**Fig. 5:**
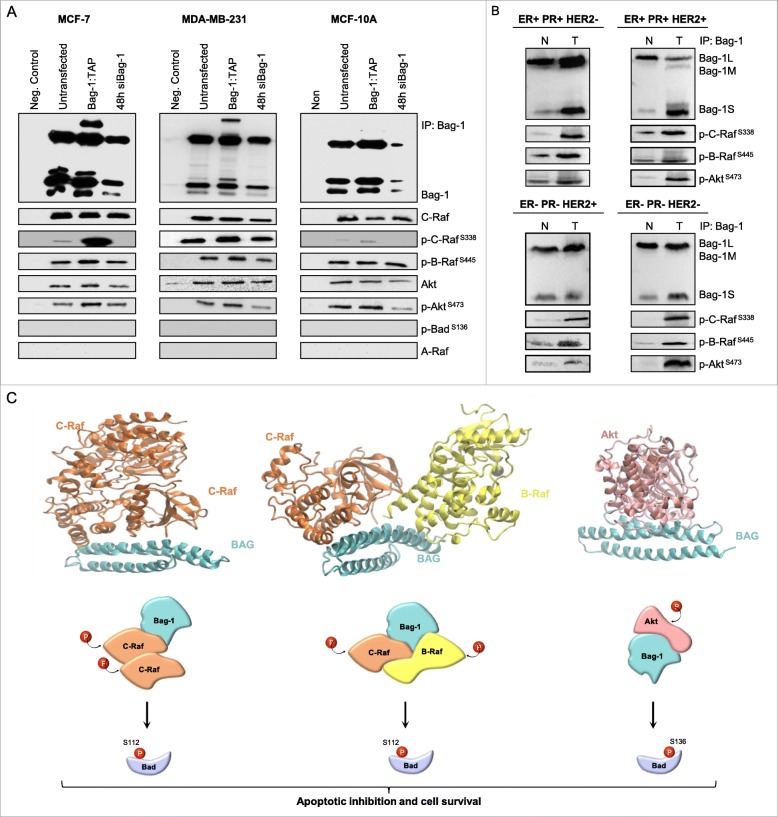
Bag-1 forms complexes with Akt and C-Raf in breast cells. **a**. MCF-7, MDA-MB-231 and MCF-10A cell lysates were immunoprecipitated (IP) with anti-Bag-1 antibody, and immunocomplexes were analyzed by immunoblots (IB) using Bag-1, C-Raf, phospho-C-Raf^Ser338^, phospho-B-Raf^Ser445^ Akt and phospho-Akt^Ser473^ antibodies. Phospho-Bad^Ser136^ and A-Raf antibodies were used as negative controls. **b**. Normal (N) and tumor (T) tissue extracts were IPed with anti-Bag-1 antibody, and immunoblots were performed using p-Akt, p-C-Raf and p-B-Raf antibodies. **c**. PRISM predictions for the complexes between Bag-1 and its interaction patners, and models for their cell survival promotion mechanism. Binding energy scores (BES) for BAG/C-Raf, BAG/C-Raf/B-Raf and BAG/Akt complexes were predicted as −30.11, −20.84, − 14.08, respectively. Bag-1 binding to C-Raf and/or B-Raf kinases activates them, which in turn phosphorylate Bad (at S112) and other downstream effectors, such as MAPK. Bag-1 binding to Akt is essential for Bad phosphorylation at S136 by the activated Akt kinase. Phosphorylated Bad inhibits apoptosis

Akt and Raf kinases were previously shown to interact directly with Bag-1 [16]. To understand whether these kinases are found in activated or non-activated states when bound to Bag-1 in breast cells, MCF-7, MDA-MB-231 and MCF-10A cell extracts were immunoprecipitated with α-Bag-1 antibody, and direct interaction partners were detected with specific antibodies using immunoblotting (Fig. 5a). We found that both phosphorylated and non-phosphorylated forms of B-Raf, C-Raf, and Akt were in direct interaction with Bag-1. These interactions were also verified in tumor samples from ER/PR + Her2-, ER/PR + Her2+, ER/PR-Her2+ and ER/PR-HER2- breast cancer patients (Fig. 5b).

We, additionally, modeled the complexes between Bag-1 and its interaction partners using the PRISM protein-protein interaction prediction tool (Fig. 5c). Predicted interactions of Bag-1 with Akt, B-Raf and C-Raf were through the BAG domain, but not the UBL domain, since only the BAG domain yielded binding energy scores that passed the threshold (BES < -10). B-Raf and C-Raf were keen to bind the BAG domain in monomeric, homodimeric or heterodimeric forms. However, binding of Raf and binding of Akt to the BAG domain were mutually exclusive, since Akt and Raf clashed when docked onto the BAG domain. Therefore, a triplet complex involving Akt/Bag-1/Raf was predicted to be unlikely.

**Fig. 6:**
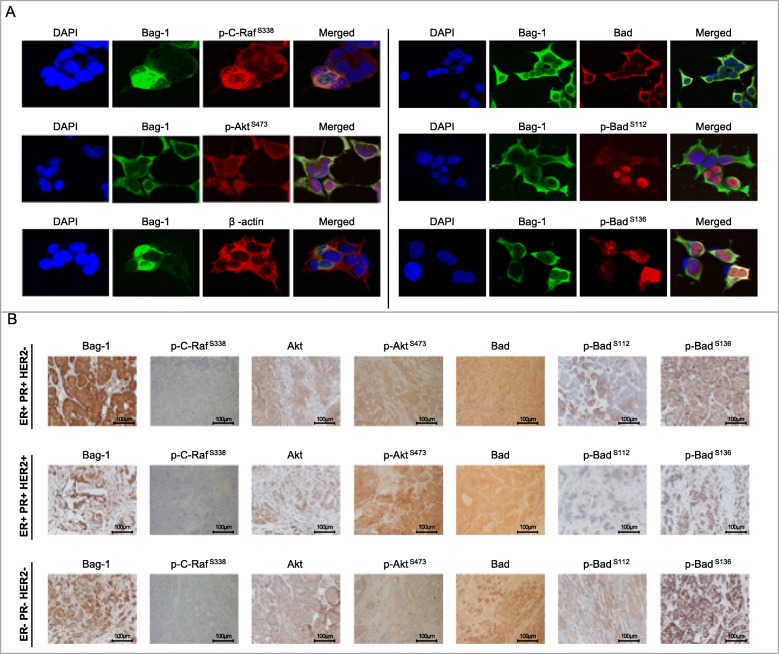
Subcellular localizations of Bag-1, Akt, C-Raf and Bad proteins in breast cancer cells. **a**. Immunocytochemical detection of Bag-1 and its pro-survival partners in MCF-7 cells. Bag-1 was stained with AlexaFlour647 goat anti-mouse (green). Phospho-Akt^Ser473^, phospho-C-Raf^Ser338^, Bad, phospho-Bad (Ser136 andSer112) as well as β-actin were stained with AlexaFlour488 goat anti-rabbit (red). Nuclei were stained with DAPI (blue). Magnification: 63X. **b**. Immunohistochemical detection of Bag-1 and its pro-survival partners in tumor tissues of breast cancer patients. Cytoplasmic and nuclear expression of Bag-1, Akt, phospho-Akt ^Ser473^, phospho-C-Raf ^Ser338^, Bad, and phospho-Bad (Ser136 andSer112) proteins were graded for patients with ER + PR + Her2-, ER + PR + Her2+ and ER-PR-Her2-subtypes. Scale bar: 100 μm

The subcellular localization of Bag-1, its interaction partners phospho-C-Raf and phospho-Akt, and their downstream effector Bad were also investigated by immune staining in breast cells and tissues (Fig. 6). Bag-1, phospho-Akt (Ser473) and phospho-C-Raf (Ser338) were localized primarily in the cytoplasm, but also in the nucleus in Bag-1 overexpressing MCF-7 cells (Fig. 6a, Additional file 1: Figure S8.). Bad was exclusively found in the cytoplasm. However, phosphorylated Bad proteins (Ser112 and Ser136) were found localized primarily nucleus in the MCF-7 cells (Fig. 6a, Additional file 1: Figure S8). The cytoplasmic and nuclear expression pattern of these proteins was also investigated in breast tumor tissues with different receptor status (Fig. 6b, Table 1). Phospho-C-Raf (S338), phospho-Akt (S473), total Bad as well as phospho-Bad Ser112 staining were observed largely in the cytoplasm, whereas phospho-Bad Ser136 expression had largely nuclear distribution. Even though, subcellular localizations of phosphorylated Bad varied between cell culture and breast tissues, these findings suggested that the nuclear localization of phospho-Bad might be effective in preventing its apoptotic function at the mitochondria.

**Table 1:** Subcellular localizations of proteins in breast cancers. n = Number of patients. Localization of Bag-1, Akt, phospho-Akt ^Ser473^, Bad, and phospho-Bad (Ser136 andSer112) proteins were graded for patients with ER + PR + Her2-, ER + PR + Her2+, ER-PR-Her2+ and ER-PR-Her2- subtypes

Tumor Subtypes	Nuclear	Cytoplasmic	Nuclear and Cytoplasmic
ER + PR + HER2-(*n* = 9)			
Bag-1	–	2	7
Akt	–	7	2
P-Akt ^S473^	–	9	–
P-Bad ^S112^	–	9	–
P-Bad ^S136^	4	–	5
Bad	–	5	–
ER + PR + HER2+(*n* = 6)			
Bag-1	–	–	6
Akt	–	5	1
P-Akt ^S473^	–	6	–
P-Bad ^S112^	–	5	–
P-Bad ^S136^	5	–	1
Bad	–	5	–
ER-PR-HER2+(*n* = 5)			
Bag-1	–	5	–
Akt	–	5	–
P-Akt ^S473^	–	5	–
P-Bad ^S112^	–	5	–
P-Bad ^S136^	3	–	2
Bad	–	2	–
ER-PR-HER2-(*n* = 5)			
Bag-1	–	2	1
Akt	–	4	–
P-Akt ^S473^	–	4	–
P-Bad ^S112^	–	3	–
P-Bad ^S136^	4	–	–
Bad	–	–	–

## Discussion

Bag-1 is an anti-apoptotic protein and is overexpressed in breast cancer [4]. Breast cancer has four major subtypes on the basis of nuclear hormone receptors (ER and PR) and Her2 expression status, and these subtypes vary in their molecular profiles, prognosis and responsiveness to chemotherapy [24, 25]. In this study, we found significantly higher Bag-1 levels in tumor tissues compared to normal tissues independent of the tumor’s receptor status. Bag-1 protein directly interacts with ER and PR [12, 26], whereas its interaction with Her2 has not been reported. Moreover, Bag-1 expression was strongly positively correlated with ER and PR expression, but negatively correlated with Her2 expression in breast tumors [21, 27–30]. Despite these differences, Bag-1 overexpression was a common feature in all breast cancer subtypes. In accordance with this finding, ectopic expression of Bag-1 increased proliferation, whereas its silencing increased apoptotic cell death in all cell lines tested independent of their receptor and tumorigenicity status [7, 31–33].

Bag-1 interacts with C-Raf, B-Raf and Akt kinases [14, 15], which are key mediators of cell survival and growth, and are frequently activated in cancer [16]. We observed significant increases in total and phosphorylated Raf levels, and phosphorylated Akt levels in tumors compared to normal tissues in breast cancer patients. Despite the differences in tissue composition between normal and tumor tissues (i.e., higher fraction of adipocytes in normal breast tissues, and higher fraction of cancerous epithelia and stroma in tumors), higher levels of Bag-1, C-Raf, B-Raf and Akt likely represented their increased expression in cancerous cells. We demonstrated that Bag-1 overexpression in MCF-7, BT-474, MDA-MB-231 and MCF-10A cells significantly increased total and phosphorylated levels of B-Raf and C-Raf as well as phosphorylated, but not total, level of Akt. Hence, Bag-1-induced effects in breast cell lines were similar to the observed alterations in breast cancer tumors. These findings further proved that Bag-1 expression is critical in breast cancer development.

We showed that Bag-1 interacted directly with Raf kinases and Akt. These interactions activate the kinases, subsequently leading to phosphorylation of Bad at S112 and S136, respectively [17]. We found Bag-1 overexpression increased Bad phosphorylation at both residues, implicating both Raf and Akt kinases in Bag-1’s anti-apoptotic function in breast cancer cells.

Targeting the interactions between Bag-1 and its binding partners with small molecules might be an effective therapeutic strategy to release inhibition of apoptosis in Bag-1 overexpressing cancer cells. To do so, the interaction surfaces must be studied at the atomic level. We modeled the interaction surfaces between Bag-1, Akt and Raf kinases, and found that Bag-1 can bind to either Akt or Raf at a time. We also modeled the interactions of Akt and Raf kinases with their small molecule inhibitors (i.e.*,* MK2206 and GW5074, data not shown). We predicted significant overlaps in the binding sites of these inhibitors and Bag-1. This finding suggests that increased Bag-1 expression in cancer cells might hinder the binding of these inhibitors to their targets. Therefore, exploiting the chemicals that disrupt Bag-1’s interactions with kinases might also increase the effectiveness of kinase inhibitors as chemotherapeutic agents.

We found that both p-Bad Ser112 and p-Bad Ser136 were localized to the nucleus whereas unphosphorylated Bad is cytoplasmic in MCF-7 cells. Previous reports suggested that Bad phosphorylation leads to its sequestration by 14–3-3 proteins in the cytoplasm [18]. Our data suggested that, nuclear sequestration of Bad upon phosphorylation might be another mechanism by which its pro-apoptotic activity at the mitochondria is prevented. In contrast to our observations in cell culture, only p-Bad Ser136 was nuclear whereas p-Bad Ser112 was exclusively cytoplasmic in breast tumor tissues. A more detailed interrogation of their subcellular localization patterns is warranted using larger numbers of patients and controlling for the clinicopathological variables such as tumor size, tumor grade, and lymph node status, age of patient, etc. Nevertheless, the distinct localization patterns of p-Bad Ser112 and p-Bad Ser136 might be meaningful given that these phosphorylation events are also mediated by distinct kinases. Studies in Bag-1 knockout mice showed that phosphorylation at Ser136, but not Ser112 or Ser155, was essential for the prevention of apoptosis in hematopoietic and neuronal cells [16]. Hence, this discrepancy between different phospho-Bad proteins might be partially due to the fact only phospho-Bad Ser136 is transported into the nucleus in vivo, as our immunohistochemistry assays showed.

## Conclusions

In conclusion, Bag-1 expression promotes cell survival and growth in breast cells and tumors. Bag-1 mediates these effects by binding and activating Akt, B-Raf and C-Raf kinases, which phosphorylate and inhibit pro-apoptotic Bad protein. Therefore, Bag-1’s interactions with Akt and Raf kinases might be targeted to prevent these survival pathways. Small molecules that disrupt these interactions might prove effective drugs in the treatment of breast cancer.

## Supplementary information

**Additional file 1: Figure S1.** Bag-1 protein expression is increased in all molecular subtypes of breast cancer. Western blots for Bag-1 in tumor and normal tissues from breast cancer patients with four major molecular subtypes; A. ER + PR + Her2-, B. ER + PR + Her2+, C. ER-PR-Her2+ D. ER-PR-Her2- breast cancer tissues. **Figure S2.** *Cell viability and apoptotic cell death following Bag-1 overexpression or Bag-1 silencing in MCF-10A cells.* XTT cell viability assay (A) and Cell Death Detection ELISAPLUS assay (B) was performed 24, 48 and 72 h after transfecting MCF-10A cells with Bag-1 expression vector, Bag-1 siRNA, and their negative controls. All values are given relative to 24 h untransfected control. Data are represented as mean ± standard error from three independent experiments for XTT assay and two independent experiments for apoptosis assay. Two-way ANOVA was used to calculate *p* values. **Figure S3.** Densitometric analysis of C-Raf, phospho-C-Raf^S338^, B-Raf, phospho-B-Raf^S445^, Akt and phospho-Akt^S473^ levels in MCF-7 (A), MDA-MB-231 (B) and MCF-10A cells (C) following Bag-1 overexpression or Bag-1 silencing. Expression levels were normalized to β-actin, and one-way ANOVA was used to assess significant changes. **Figure S4.** Western blots for C-Raf and phospho-C-Raf in tumor and normal tissues from breast cancer patients with four major molecular subtypes; A, ER + PR + Her2-, B. ER + PR + Her2+, C. ER-PR-Her2+ D. ER-PR-Her2- breast cancer tissues. **Figure S5.** Western blots for B-Raf and phospho-B-Raf in tumor and normal tissues from breast cancer patients with four major molecular subtypes; A. ER + PR + Her2-, B. ER + PR + Her2+, C. ER-PR-Her2+ D. ER-PR-Her2- breast cancer patients. **Figure S6.** Densitometric analysis of Bad, phospho-Bad^S136^, phospho-Bad^S112^ and 14–3-3 protein levels in MCF-7 and MDA-MB-231 cells following Bag-1 overexpression or Bag-1 silencing. **Figure S7.** Effects of GW5074 and MK2226 on C-Raf, Akt and Bad phosphorylation levels in MCF-7 and MDA-MB-231 cells. A. Immunoblot analysis of total C-Raf, phosphorylated C-Raf and phosphorylated Bad levels in cells treated with C-Raf inhibitor GW5074. B. Immunoblot analysis of total Akt, phosphorylated Akt and phosphorylated Bad levels in cells treated with Akt inhibitor MK2226. β-actin was used as a loading control. **Figure S8.** Quantitative analysis for colocalization of Bag-1 with Akt, C-Raf and Bad proteins in MCF-7 cells. Pearson’s *r* was calculated from 3 images using green (Bag-1) and red (other proteins) channels in Fiji plug-in of ImageJ. Data are presented as mean ± std. (*p* < 0.001 for all correlations).

## Data Availability

The datasets used and analysed during this study are available from the corresponding author on reasonable request.

## Abbreviations

Bag-1: Bcl-2-associated athanogene 1
ER: Estrogen receptor
HER2: Human epidermal growth factor receptor 2
ICC: Immunocytochemistry
IHC: Immunohistochemistry
MAPKs: Mitogen-activated protein kinases
NLS: Nuclear localization signal
PR: Progesterone receptor
UBL: Ubiquitin-like
