# Supplementary material for: Bag-1 stimulates Bad phosphorylation through activation of Akt and Raf kinases to mediate cell survival in breast cancer
Source: BMC Cancer. 2019 Dec 28;19:1254. doi: 10.1186/s12885-019-6477-4 (PMC6935482; doi:10.1186/s12885-019-6477-4)
Supplement: Supplementary file 1 — Additional file 1: Figure S1. Bag-1 protein expression is increased in all molecular subtypes of breast cancer. Western blots for Bag-1 in tumor and normal tissues from breast cancer patients with four major molecular subtypes; A. ER + PR + Her2-, B. ER + PR + Her2+, C. ER-PR-Her2+ D. ER-PR-Her2- breast cancer tissues. Figure S2. Cell viability and apoptotic cell death following Bag-1 overexpression or Bag-1 silencing in MCF-10A cells. XTT cell viability assay (A) and Cell Death Detection ELISAPLUS assay (B) was performed 24, 48 and 72 h after transfecting MCF-10A cells with Bag-1 expression vector, Bag-1 siRNA, and their negative controls. All values are given relative to 24 h untransfected control. Data are represented as mean ± standard error from three independent experiments for XTT assay and two independent experiments for apoptosis assay. Two-way ANOVA was used to calculate p values. Figure S3. Densitometric analysis of C-Raf, phospho-C-RafS338, B-Raf, phospho-B-RafS445, Akt and phospho-AktS473 levels in MCF-7 (A), MDA-MB-231 (B) and MCF-10A cells (C) following Bag-1 overexpression or Bag-1 silencing. Expression levels were normalized to β-actin, and one-way ANOVA was used to assess significant changes. Figure S4. Western blots for C-Raf and phospho-C-Raf in tumor and normal tissues from breast cancer patients with four major molecular subtypes; A, ER + PR + Her2-, B. ER + PR + Her2+, C. ER-PR-Her2+ D. ER-PR-Her2- breast cancer tissues. Figure S5. Western blots for B-Raf and phospho-B-Raf in tumor and normal tissues from breast cancer patients with four major molecular subtypes; A. ER + PR + Her2-, B. ER + PR + Her2+, C. ER-PR-Her2+ D. ER-PR-Her2- breast cancer patients. Figure S6. Densitometric analysis of Bad, phospho-BadS136, phospho-BadS112 and 14–3-3 protein levels in MCF-7 and MDA-MB-231 cells following Bag-1 overexpression or Bag-1 silencing. Figure S7. Effects of GW5074 and MK2226 on C-Raf, Akt and Bad phosphorylation levels in MCF-7 and MDA-MB-231 ce [file 12885_2019_6477_MOESM1_ESM.pptx]

## Slide 1
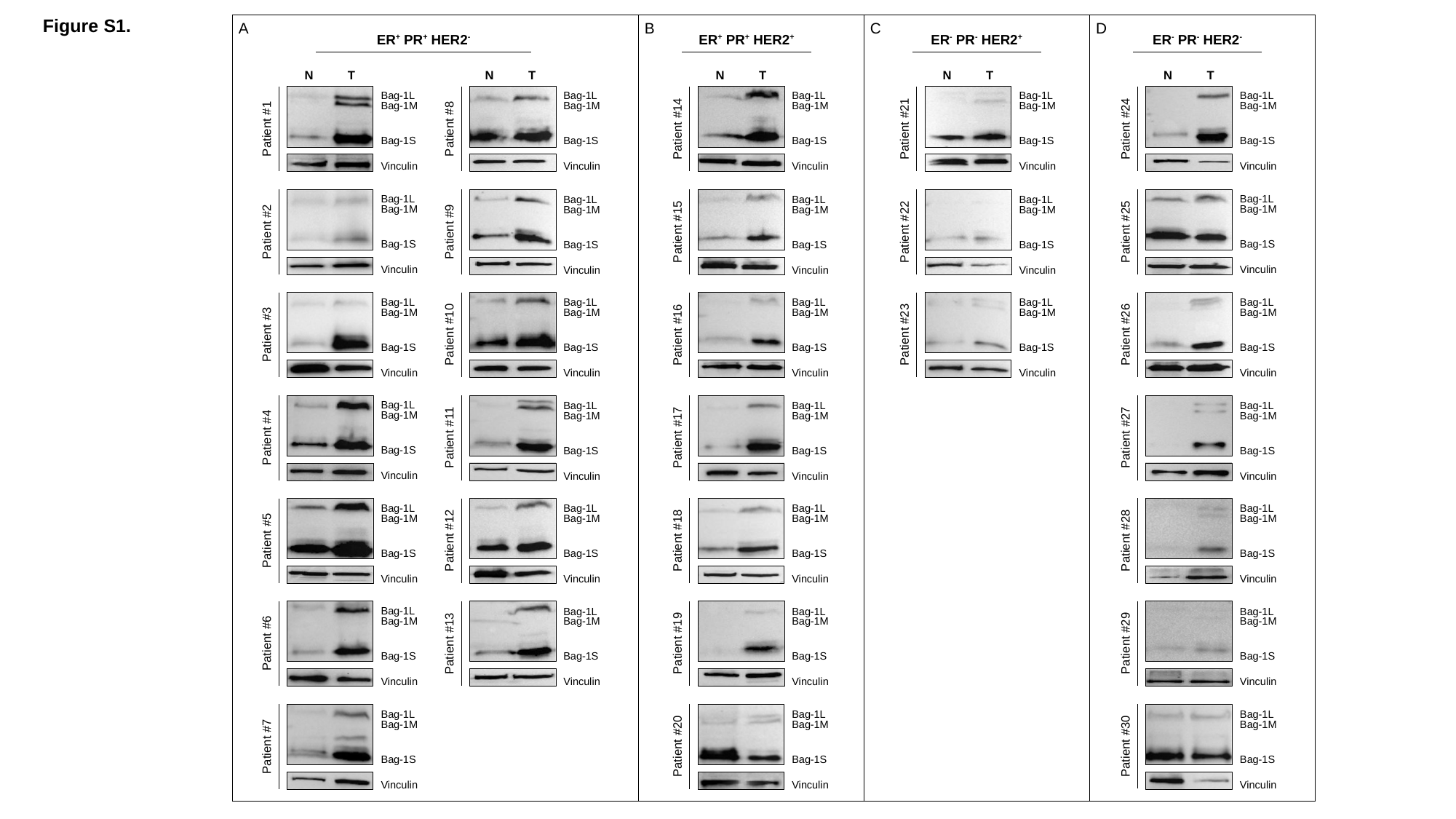

Figure S1.
A
B
C
D
ER+ PR+ HER2-
ER+ PR+ HER2+
ER- PR- HER2+
ER- PR- HER2-
N T
N T
Bag-1L
Bag-1M
Bag-1S
Vinculin
Bag-1L
Bag-1M
Bag-1S
Vinculin
Patient #1
Patient #2
Patient #3
Patient #4
Patient #5
Patient #6
Patient #7
Patient #8
Patient #9
Patient #10
Patient #11
Patient #12
Patient #13
Bag-1L
Bag-1M
Bag-1S
Vinculin
Bag-1L
Bag-1M
Bag-1S
Vinculin
Bag-1L
Bag-1M
Bag-1S
Vinculin
Bag-1L
Bag-1M
Bag-1S
Vinculin
Bag-1L
Bag-1M
Bag-1S
Vinculin
Bag-1L
Bag-1M
Bag-1S
Vinculin
Bag-1L
Bag-1M
Bag-1S
Vinculin
Bag-1L
Bag-1M
Bag-1S
Vinculin
Bag-1L
Bag-1M
Bag-1S
Vinculin
Bag-1L
Bag-1M
Bag-1S
Vinculin
Bag-1L
Bag-1M
Bag-1S
Vinculin
N T
Bag-1L
Bag-1M
Bag-1S
Vinculin
Patient #14
Patient #15
Patient #16
Patient #17
Bag-1L
Bag-1M
Bag-1S
Vinculin
Bag-1L
Bag-1M
Bag-1S
Vinculin
Bag-1L
Bag-1M
Bag-1S
Vinculin
Patient #18
Patient #19
Patient #20
Bag-1L
Bag-1M
Bag-1S
Vinculin
Bag-1L
Bag-1M
Bag-1S
Vinculin
Bag-1L
Bag-1M
Bag-1S
Vinculin
N T
Bag-1L
Bag-1M
Bag-1S
Vinculin
Patient #21
Patient #22
Patient #23
Bag-1L
Bag-1M
Bag-1S
Vinculin
Bag-1L
Bag-1M
Bag-1S
Vinculin
N T
Bag-1L
Bag-1M
Bag-1S
Vinculin
Patient #24
Patient #25
Patient #26
Patient #27
Bag-1L
Bag-1M
Bag-1S
Vinculin
Bag-1L
Bag-1M
Bag-1S
Vinculin
Bag-1L
Bag-1M
Bag-1S
Vinculin
Patient #28
Patient #29
Patient #30
Bag-1L
Bag-1M
Bag-1S
Vinculin
Bag-1L
Bag-1M
Bag-1S
Vinculin
Bag-1L
Bag-1M
Bag-1S
Vinculin

## Slide 2
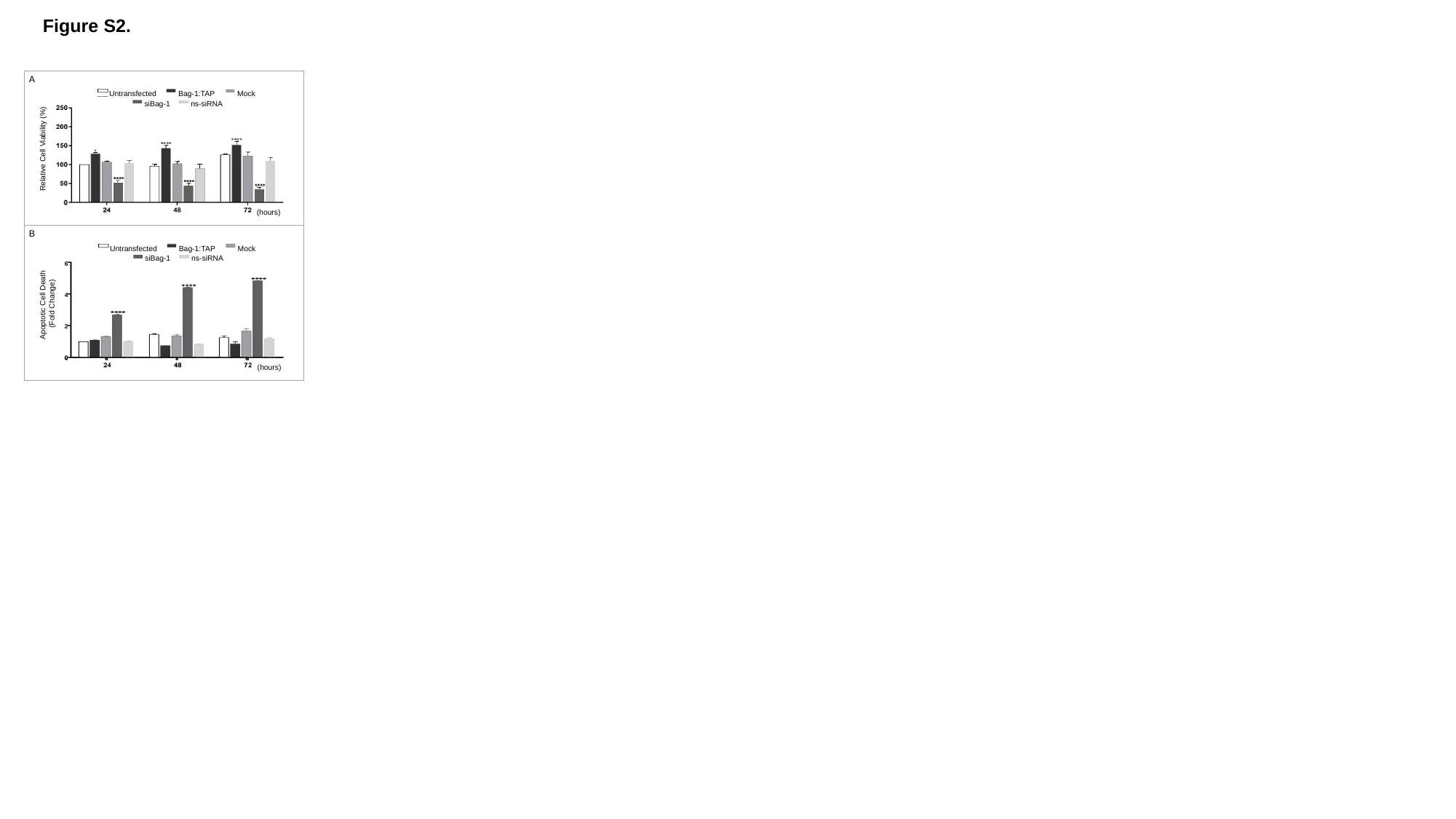

Suppl 1.
Figure S2.
A
B
Untransfected
Bag-1:TAP
Mock
siBag-1
ns-siRNA
Relative Cell Viability (%)
(hours)
Untransfected
Bag-1:TAP
Mock
siBag-1
ns-siRNA
Apoptotic Cell Death (Fold Change)
6
4
2
0
(hours)

## Slide 3
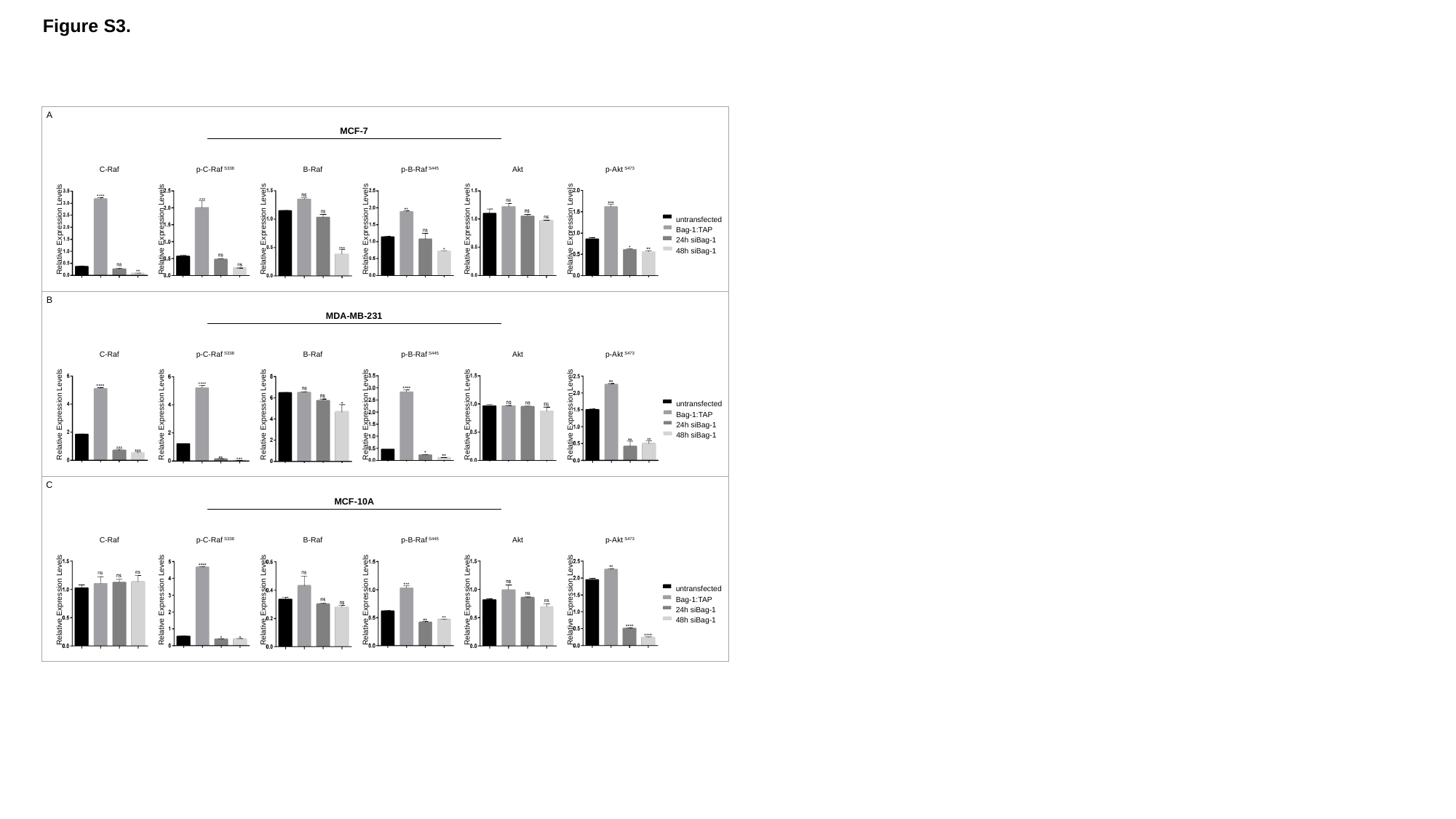

Figure S3.
A
B
C
MCF-7
C-Raf
p-C-Raf S338
B-Raf
p-B-Raf S445
Akt
p-Akt S473
Relative Expression Levels
Relative Expression Levels
Relative Expression Levels
Relative Expression Levels
Relative Expression Levels
Relative Expression Levels
untransfected
Bag-1:TAP
24h siBag-1
48h siBag-1
MDA-MB-231
C-Raf
p-C-Raf S338
B-Raf
p-B-Raf S445
Akt
p-Akt S473
Relative Expression Levels
Relative Expression Levels
Relative Expression Levels
Relative Expression Levels
Relative Expression Levels
Relative Expression Levels
untransfected
Bag-1:TAP
24h siBag-1
48h siBag-1
MCF-10A
C-Raf
p-C-Raf S338
B-Raf
p-B-Raf S445
Akt
p-Akt S473
Relative Expression Levels
Relative Expression Levels
Relative Expression Levels
Relative Expression Levels
Relative Expression Levels
Relative Expression Levels
untransfected
Bag-1:TAP
24h siBag-1
48h siBag-1

## Slide 4
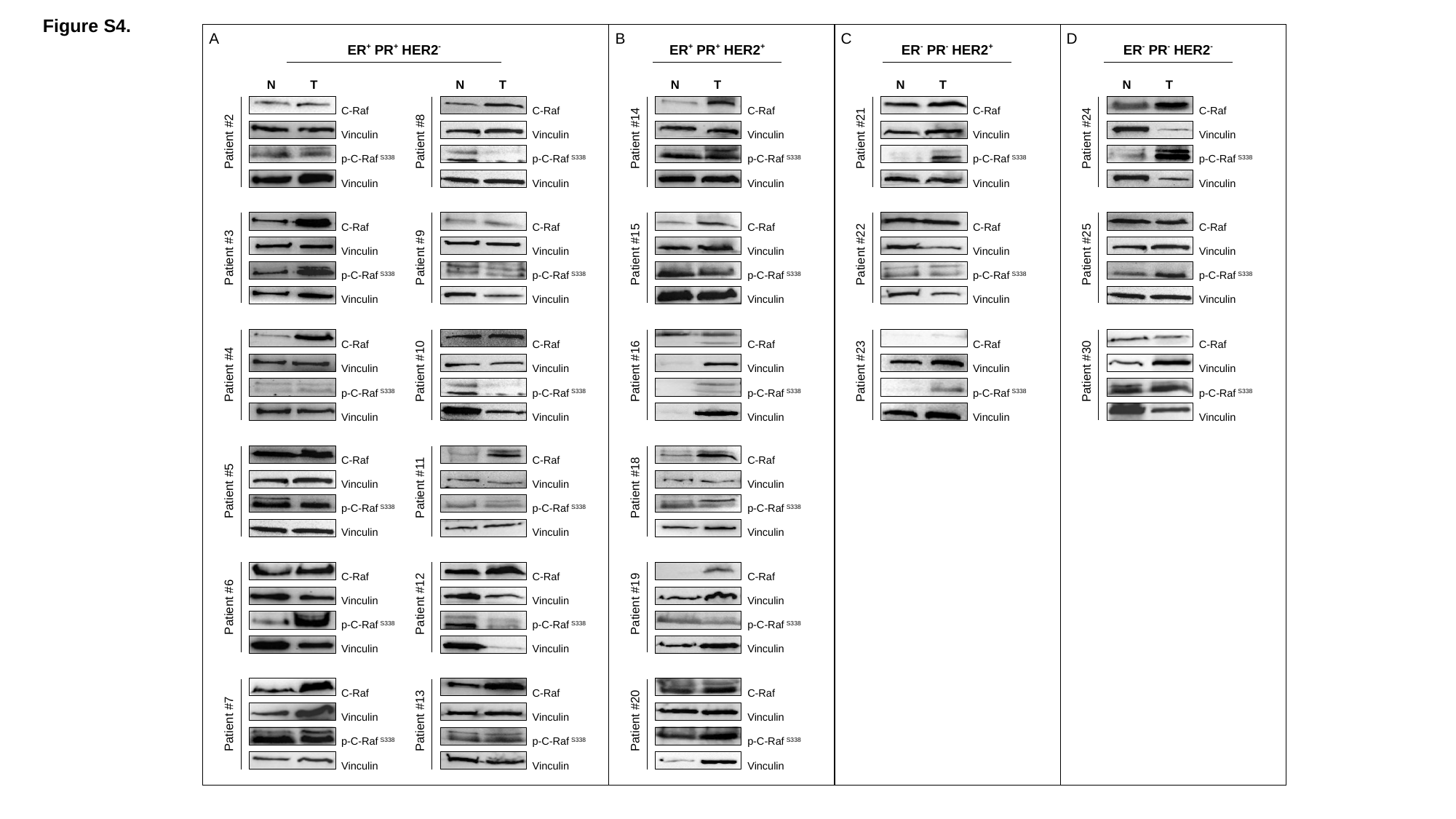

Figure S4.
A
B
C
D
ER+ PR+ HER2-
ER+ PR+ HER2+
ER- PR- HER2+
ER- PR- HER2-
N T
N T
C-Raf
Vinculin
p-C-Raf S338
Vinculin
C-Raf
Vinculin
p-C-Raf S338
Vinculin
C-Raf
Vinculin
p-C-Raf S338
Vinculin
C-Raf
Vinculin
p-C-Raf S338
Vinculin
C-Raf
Vinculin
p-C-Raf S338
Vinculin
C-Raf
Vinculin
p-C-Raf S338
Vinculin
C-Raf
Vinculin
p-C-Raf S338
Vinculin
Patient #8
Patient #9
Patient #10
Patient #11
Patient #12
Patient #13
Patient #2
C-Raf
Vinculin
p-C-Raf S338
Vinculin
Patient #3
C-Raf
Vinculin
p-C-Raf S338
Vinculin
Patient #4
C-Raf
Vinculin
p-C-Raf S338
Vinculin
Patient #5
Patient #6
Patient #7
C-Raf
Vinculin
p-C-Raf S338
Vinculin
C-Raf
Vinculin
p-C-Raf S338
Vinculin
N T
C-Raf
Vinculin
p-C-Raf S338
Vinculin
C-Raf
Vinculin
p-C-Raf S338
Vinculin
C-Raf
Vinculin
p-C-Raf S338
Vinculin
C-Raf
Vinculin
p-C-Raf S338
Vinculin
C-Raf
Vinculin
p-C-Raf S338
Vinculin
C-Raf
Vinculin
p-C-Raf S338
Vinculin
Patient #14
Patient #15
Patient #16
Patient #18
Patient #19
Patient #20
N T
C-Raf
Vinculin
p-C-Raf S338
Vinculin
C-Raf
Vinculin
p-C-Raf S338
Vinculin
C-Raf
Vinculin
p-C-Raf S338
Vinculin
Patient #21
Patient #22
Patient #23
N T
C-Raf
Vinculin
p-C-Raf S338
Vinculin
C-Raf
Vinculin
p-C-Raf S338
Vinculin
C-Raf
Vinculin
p-C-Raf S338
Vinculin
Patient #24
Patient #25
Patient #30

## Slide 5
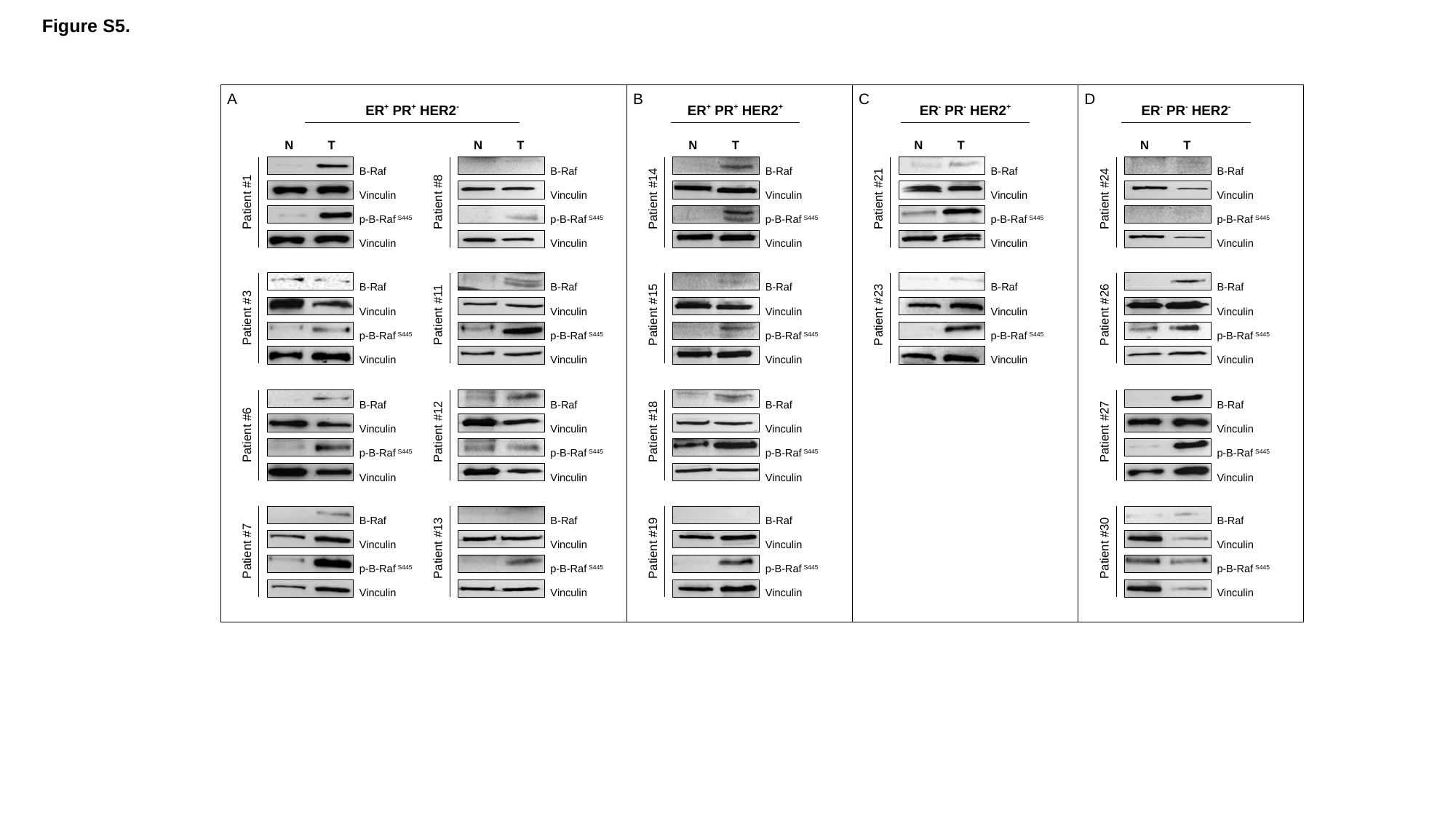

Figure S5.
A
B
C
D
ER+ PR+ HER2-
ER+ PR+ HER2+
ER- PR- HER2+
ER- PR- HER2-
N T
N T
N T
N T
N T
B-Raf
Vinculin
p-B-Raf S445
Vinculin
B-Raf
Vinculin
p-B-Raf S445
Vinculin
B-Raf
Vinculin
p-B-Raf S445
Vinculin
B-Raf
Vinculin
p-B-Raf S445
Vinculin
B-Raf
Vinculin
p-B-Raf S445
Vinculin
Patient #14
Patient #21
Patient #24
Patient #1
Patient #8
B-Raf
Vinculin
p-B-Raf S445
Vinculin
B-Raf
Vinculin
p-B-Raf S445
Vinculin
B-Raf
Vinculin
p-B-Raf S445
Vinculin
B-Raf
Vinculin
p-B-Raf S445
Vinculin
B-Raf
Vinculin
p-B-Raf S445
Vinculin
Patient #11
Patient #15
Patient #23
Patient #26
Patient #3
B-Raf
Vinculin
p-B-Raf S445
Vinculin
B-Raf
Vinculin
p-B-Raf S445
Vinculin
B-Raf
Vinculin
p-B-Raf S445
Vinculin
B-Raf
Vinculin
p-B-Raf S445
Vinculin
Patient #12
Patient #18
Patient #27
Patient #6
B-Raf
Vinculin
p-B-Raf S445
Vinculin
B-Raf
Vinculin
p-B-Raf S445
Vinculin
B-Raf
Vinculin
p-B-Raf S445
Vinculin
B-Raf
Vinculin
p-B-Raf S445
Vinculin
Patient #13
Patient #19
Patient #30
Patient #7

## Slide 6
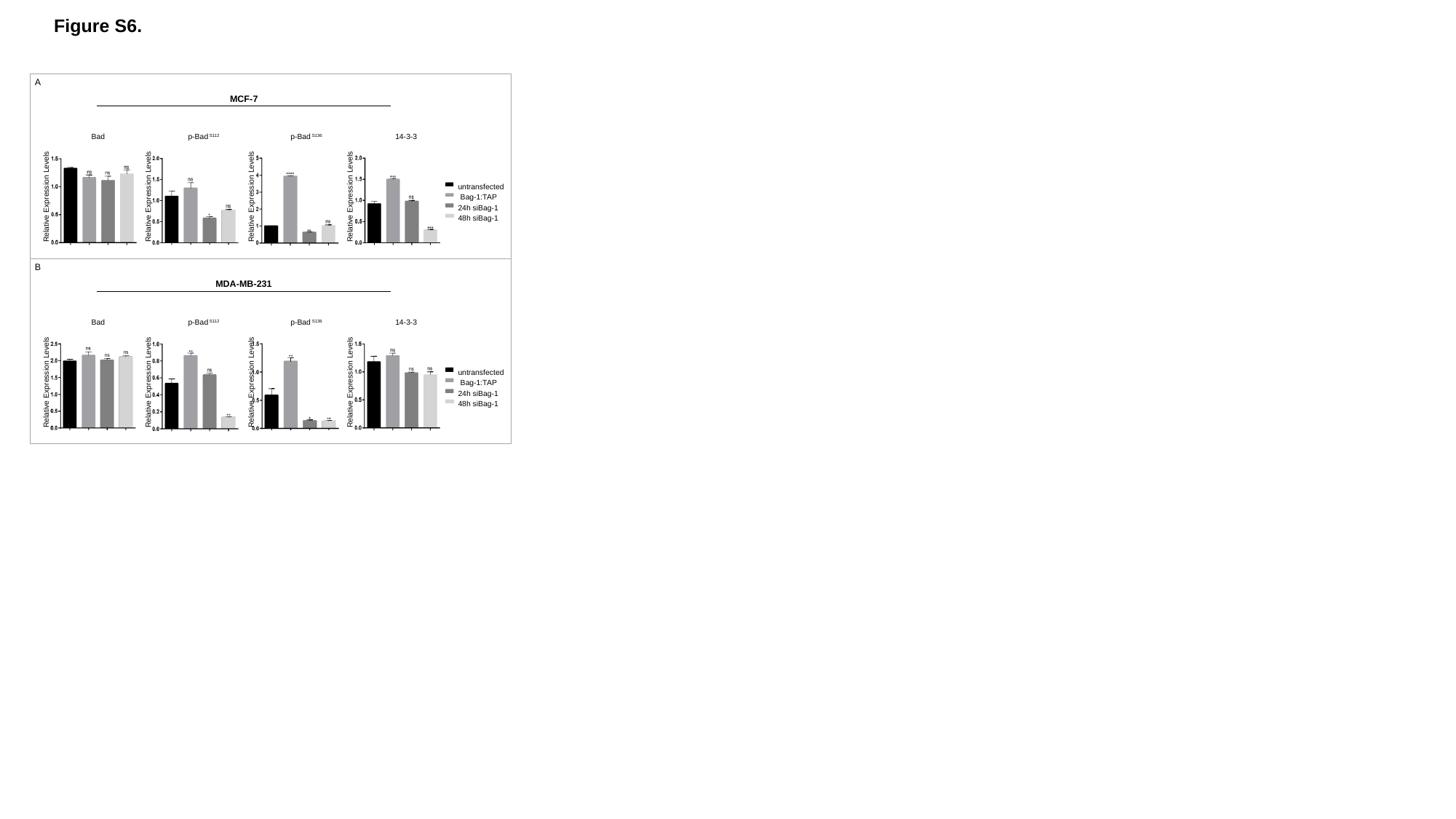

Figure S6.
A
B
MCF-7
Bad
p-Bad S112
p-Bad S136
14-3-3
untransfected
Bag-1:TAP
24h siBag-1
48h siBag-1
Relative Expression Levels
Relative Expression Levels
Relative Expression Levels
Relative Expression Levels
MDA-MB-231
Bad
p-Bad S112
p-Bad S136
14-3-3
untransfected
Bag-1:TAP
24h siBag-1
48h siBag-1
Relative Expression Levels
Relative Expression Levels
Relative Expression Levels
Relative Expression Levels

## Slide 7
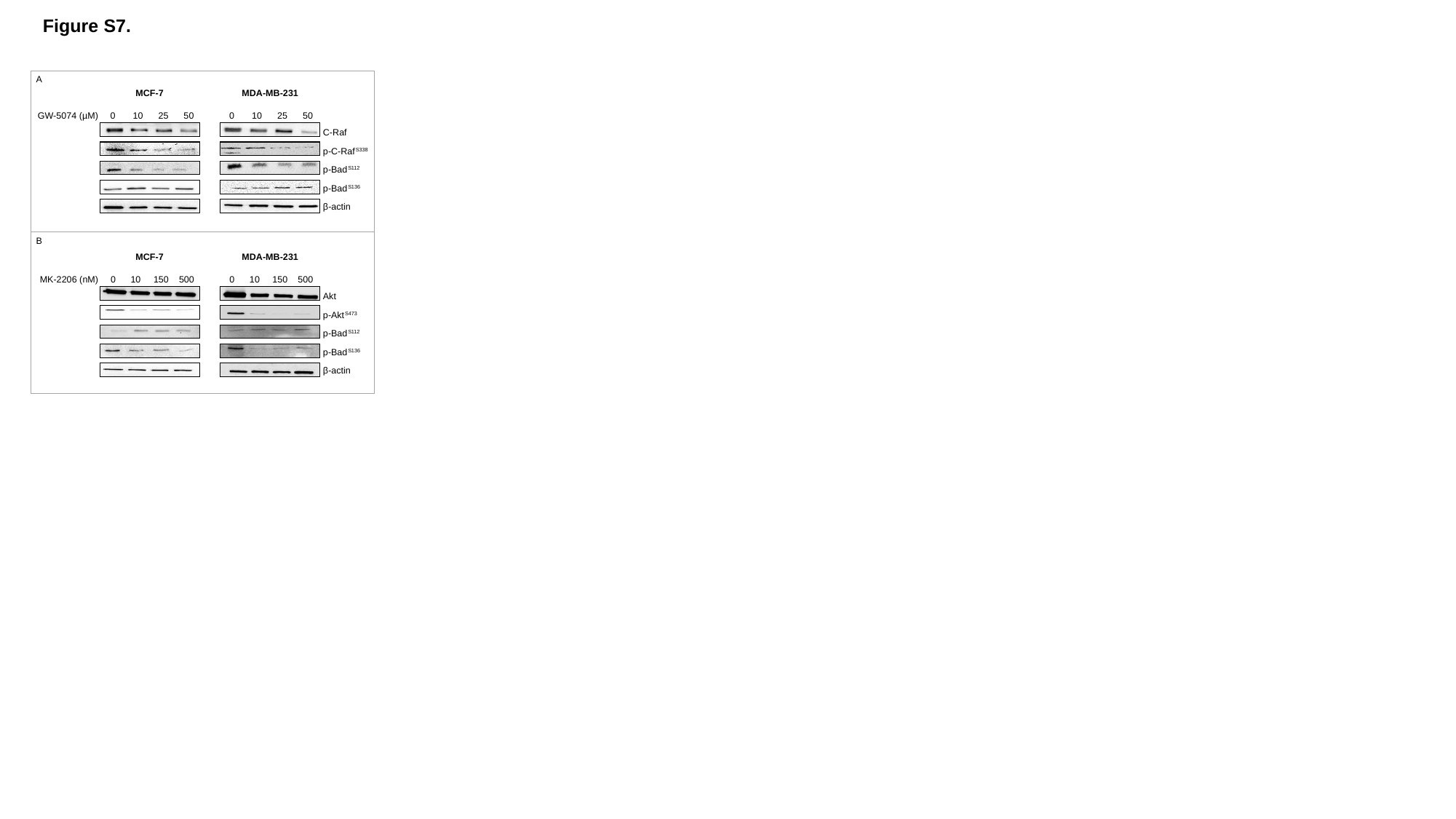

Figure S7.
A
B
MCF-7
MDA-MB-231
GW-5074 (µM)
0 10 25 50
0 10 25 50
C-Raf
p-C-Raf S338
p-Bad S112
p-Bad S136
β-actin
MCF-7
MDA-MB-231
MK-2206 (nM)
0 10 150 500
0 10 150 500
Akt
p-Akt S473
p-Bad S112
p-Bad S136
β-actin

## Slide 8
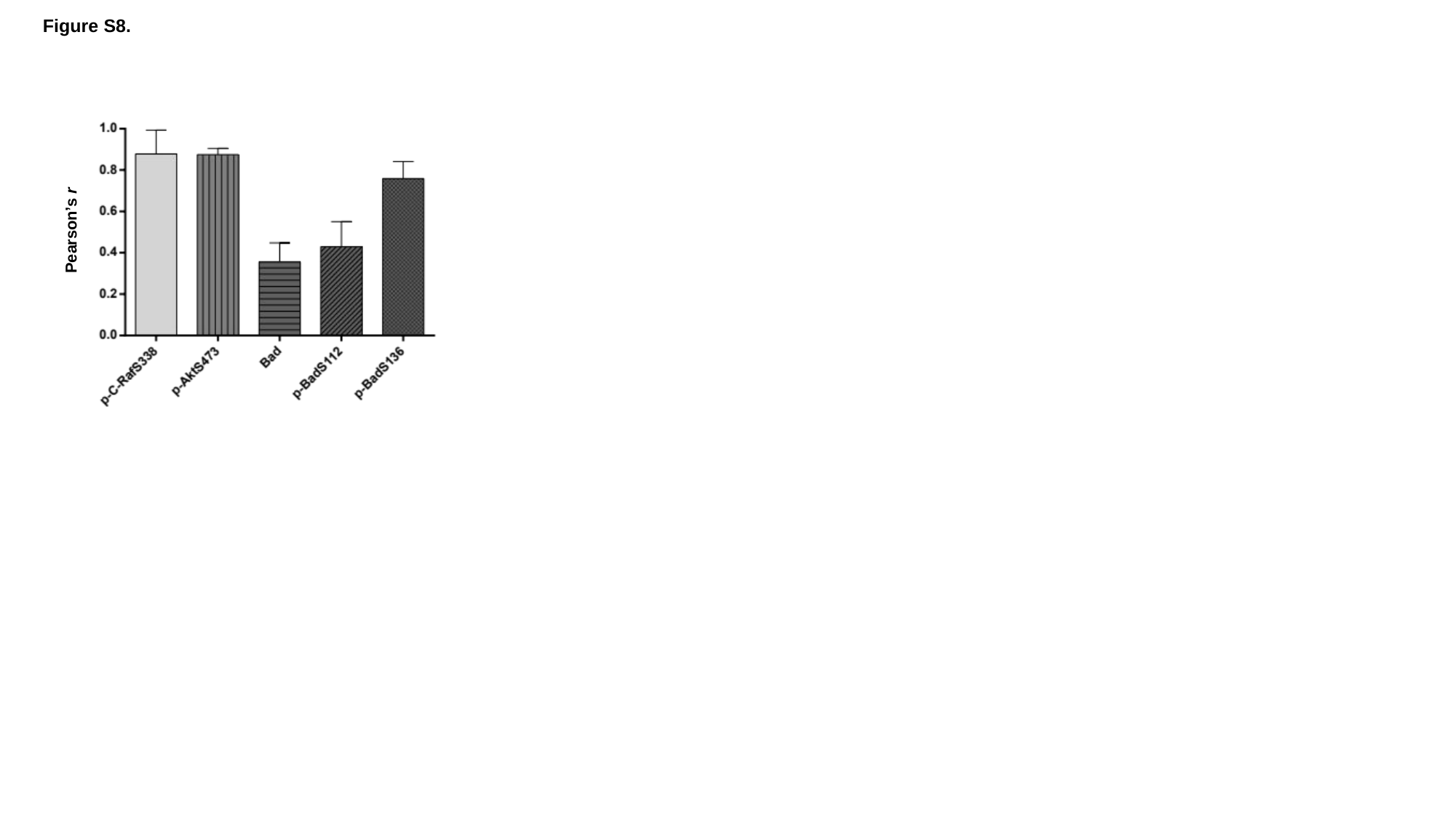

Figure S8.
Pearson’s r
